# Ocular Metastatic Tumor in a Patient With Hepatocellular Carcinoma

**DOI:** 10.7759/cureus.74435

**Published:** 2024-11-25

**Authors:** Takuma Iwai, Hiroshi Makino, Hiroshi Yoshida

**Affiliations:** 1 Surgery, Nippon Medical School, Tokyo, JPN; 2 Oncology, Nippon Medical School, Tokyo, JPN

**Keywords:** eye-ball metastases, hepatocellular carcinoma (hcc), ocular metastases, pain control, radiation therapy

## Abstract

While orbital floor metastasis from hepatocellular carcinoma (HCC) has been reported, ocular (eyeball) metastasis is exceedingly rare. Most ocular metastases originate from breast or lung cancer. In this article, we present the case of a 65-year-old man diagnosed with HCC with central necrosis (cT3N0M0, stage III) based on characteristic imaging findings. As liver resection was not indicated, biliary drainage, ascites control, and transcatheter arterial chemoembolization (TACE) were performed. On day 62 of admission, he developed right-sided visual disturbance accompanied by severe pain. A fundus examination and head CT scan by an ophthalmologist indicated a tumor in the right eye. A whole-body CT scan was performed again, revealing no other distant metastases apart from the eye. Based on the clinical course, ocular metastasis from HCC was diagnosed. Due to poor general condition, enucleation was not performed. Pain management with medication alone was insufficient, but two sessions of radiation therapy significantly alleviated the pain.

Prompt treatment is crucial as ocular metastasis is associated with pain and vision impairment, significantly affecting the patient's quality of life. When systemic treatment is not feasible, local treatment options, such as radiation therapy, become important. Radiation therapy for ocular metastases is valuable, as it can provide rapid symptomatic relief.

## Introduction

While orbital floor metastases from hepatocellular carcinoma (HCC) have occasionally been reported, ocular (eyeball) metastases remain extremely rare, with most ocular metastases originating from lung or breast cancer [1,2]. HCC typically progresses through local invasion and vascular spread, with common metastatic sites including the lungs, bones, and adrenal glands [3]. Ocular metastasis is an unexpected event, as the ocular region is not a frequent target for metastatic cancer cells [3]. In HCC patients, distant metastases typically indicate a terminal stage, often associated with multiple comorbidities and poor performance status [4]. Due to the advanced nature of the disease and the patients' limited tolerance, systemic therapy is usually not a feasible option for those with advanced HCC [5]. However, ocular metastases can significantly impact the quality of life, frequently causing pain and visual impairment, underscoring the importance of prompt symptomatic treatment [6]. Here, we report a case of ocular metastasis during the treatment for HCC.

## Case presentation

A 69-year-old man was admitted to our hospital with fatigue and abdominal distension. He had no significant medical history but reported a history of chronic alcohol consumption, approximately 50 grams per day for 20 years. Initial laboratory values (Table 1) were as follows: white blood cell count, 5,120/µL; serum hemoglobin concentration, 13.9 g/dL; serum platelet count, 8.2 x 10^4^/µL; serum total bilirubin, 3.1 mg/dL; serum direct bilirubin, 2.1 mg/dL; serum aspartate aminotransferase, 102 U/L; serum alanine aminotransferase, 52 U/L; serum lactate dehydrogenase, 271 U/L; serum alkaline phosphatase, 1,023 U/L; gamma-glutamyl transpeptidase, 1,772 U/L; serum albumin, 3.6 g/dl; prothrombin time, 50.2%; protein induced by vitamin K absence-II (PIVKA-II), 7652 mU/mL; and α-fetoprotein (AFP) 56 ng/mL. Hepatitis B surface antigen and hepatitis C virus (HCV) antibodies were both negative.

**Table 1:** Clinical data on admission WBC: White blood cell count; Hb: Hemoglobin concentration; PLT: Serum platelet count; T-Bil: Total bilirubin; D-Bil: Direct bilirubin; GOT: Aspartate aminotransferase; GPT: Alanine aminotransferase; LDH: Lactate dehydrogenase; ALP: Alkaline phosphatase; γGTP: Gamma-glutamyl transpeptidase; ALB: Serum albumin; PT: Prothrombin time; PIVKA-Ⅱ: Protein induced by vitamin K absence-II; AFP: α-fetoprotein; APTT: Activated partial thromboplastin time.

Parameters	Result	Unit
WBC	5,120	/μl
RBC	406	× 10^4^/μl
Hb	13.9	g/dl
Ht	41.5	%
PLT	8.2	× 10^4^/μl
TP	7.2	g/dl
ALB	3.6	g/dl
BUN	10	mg/dl
CRE	0.52	mg/dl
Na	141	mEq/l
K	3.6	mEq/l
Cl	106	mEq/l
T-Bil	3.1	mg/dl
D-Bil	2.1	mg/dl
GOT	102	U/l
GPT	52	U/l
LDH	271	U/l
ALB	3.6	g/dl
ALP	1,023	U/l
γ-GTP	1,772	U/l
CK	96	U/l
AMY	58	U/l
CRP	0.03	mg/dl
AFP	56	ng/ml
PIVKA-Ⅱ	7,652	mAU/ml
PT	50.2	%
APTT	34.8	sec

**Figure 1:**
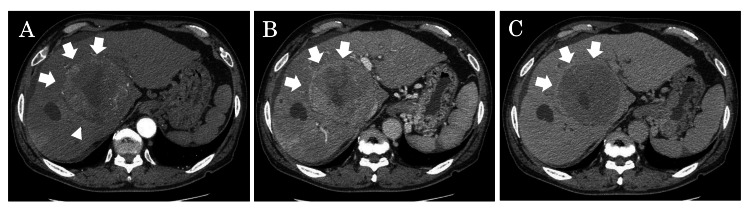
Abdominal CT findings CT showed a mass occupying the medial hepatic zone to the right lobe, with early dark staining in the contrast-enhanced arterial phase and washout in the portal venous phase (white arrow). (A) Arterial phase; (B) portal phase; and (C) delay phase. The central part of the mass showed a heterogeneous low-absorption image, suggesting HCC with necrosis. In addition, enlarged lymph nodes around the aorta were observed. HCC: Hepatocellular carcinoma.

Dynamic CT revealed a hypervascular tumor with washout (Figure 1), and magnetic resonance imaging (MRI) showed washout in all regions except the center, along with high signal intensity on diffusion-weighted images.

We diagnosed hepatocellular carcinoma with central necrosis. The patient had cirrhosis (Child-Pugh classification B) and portal hypertension, and the clinical stage of the HCC was cT3N0M0 stage III.

**Figure 2:**
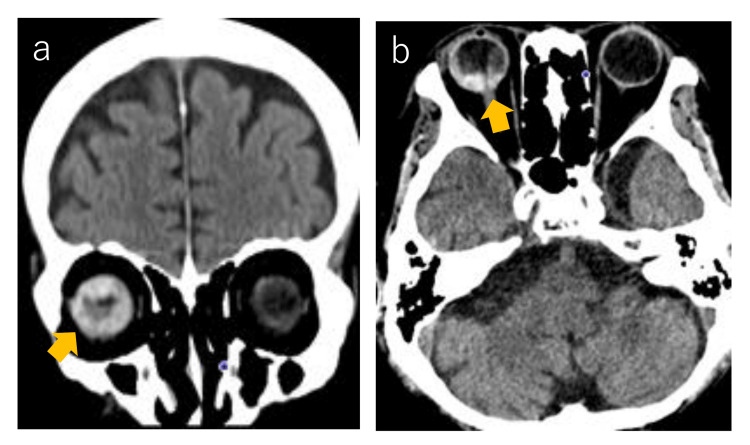
Brain CT findings Coronal (a) and axial (b) sections of the CT are shown. A highly absorbing mass was found in the right eyeball (yellow arrow). Whole-body CT images showed no apparent distant metastases other than the ocular tumor.

As surgery was not indicated, the patient was treated symptomatically (biliary drainage, ascites control, and transcatheter arterial chemoembolization). On the 62nd day of admission, the patient developed right-sided visual impairment accompanied by severe pain. Fundus examination and head CT scan conducted by an ophthalmologist revealed a tumor in the right eye (Figure 2, indicated by the arrow).

A whole-body CT scan showed no other distant metastases. Based on the clinical course, ocular metastasis of hepatocellular carcinoma was suspected. Enucleation was not possible due to the patient's poor general condition. Opioids, steroids, and mannitol were administered but did not relieve the pain. However, after two rounds (10 Gy) of ocular irradiation, the pain was significantly reduced. The patient died 10 days later due to intra-abdominal hemorrhage caused by hepatocellular carcinoma, although his pain remained well controlled during this time. It should be noted again that the symptoms that led to the diagnosis in this case were acute visual disturbances, eye pain, and inexplicable head distress.

## Discussion

Ocular metastases are estimated to account for 70% of cases originating from breast and lung cancer [1,2]. While scattered reports exist of orbital floor metastases associated with hepatocellular carcinoma, ocular metastases are extremely rare. Since ocular metastases often cause pain and visual impairment, the treatment strategy can significantly impact the patient's quality of life [5]. Therefore, prompt symptomatic treatment is essential. Systemic therapy may be an option if the primary cancer is amenable to chemotherapy, as is the case with lung cancer; otherwise, local treatment should be prioritized [6]. Considering the patient's overall condition, primary cancer type, and prognosis, treatment plans should be collaboratively discussed with ophthalmologists and radiologists to ensure optimal management. HCC’s metastatic behavior is influenced by its high vascularity and its tendency to spread hematogenously [3]. However, ocular metastasis may occur under specific circumstances, particularly when systemic conditions allow cancer cells to bypass typical metastatic pathways. Further research is needed to clarify these mechanisms.

A limitation of this case is the lack of histopathological diagnosis, but the treatment strategy was discussed in the cancer committee of our institution with surgeons, internists, radiologists, pathologists, ophthalmologists, and anesthetists. A biopsy of the tumor was considered to have a high risk of dissemination and bleeding, and treatment was planned based on the clinical diagnosis. Enucleation was difficult in terms of surgical tolerance. Reported cases of orbital floor metastasis of hepatocellular carcinoma are shown in Table 2 [8-32].

**Table 2:** Orbital floor metastasis in HCC cases RT: Radiation therapy; ND: Not described; HCC: Hepatocellular carcinoma.

Year	Author [Reference number]	Sex	Age (y/o)	Treatment	Survival
1980	Lubin et al. [8]	M	69	RT (30 Gy)	ND
1981	Zubler et al. [9]	M	64	RT (40 Gy) + chemotherapy	3 months
1990	Wakisaka et al. [10]	M	58	Craniotomy	11 months
1992	Phanthumchinda et al. [11]	F	29	RT (54 Gy)	ND
1994	Loo et al. [12]	F	71	Orbitotomy	3 months
1994	Schwab et al. [13]	M	19	Orbitotomy	2 weeks
1994	Tranfa et al. [14]	M	85	Orbitotomy	ND
1998	Font et al. [15]	F	79	RT (Gy unknown)	3 years
1999	Scolyer et al. [16]	M	77	None	ND
2000	Kim et al. [17]	F	56	None	2 months
2005	Gupta et al. [18]	M	45	None	ND
2006	Machado-Netto et al. [19]	M	57	Chemotherapy	15 months
2006	Oida et al. [20]	M	72	Frontotemporal craniotomy	4 months
2007	Srinivasan et al. [21]	F	76	None	ND
2008	Hirunwiwatkul et al. [22]	F	74	NA	2 months
2008	Fonseca Júnior et al. [23]	M	57	Biopsy only	15 months
2009	Quick et al. [24]	M	58	RT (58Gy)	20 months
2011	Mustapha and Madachi [25]	F	25	ND	ND
2011	Guerriero et al. [26]	M	45	Chemotherapy, RT	ND
2014	Eldesouky et al. [27]	M (6 cases)	47-70	OP, chemotherapy, (1 case) RT	10.2 months (mean)
2017	Geske et al. [28]	M	ND	Biopsy only	ND
2020	Protopapa et al. [29]	M	ND	RT	ND
2021	Baldovin et al. [30]	M	62	Orbitotomy	ND
2021	Rana et al. [31]	M	66	RT, Chemotherapy	ND
2021	Filippini et al. [32]	M	71	Pain control	3 months

As there are no reported cases of ocular metastases of hepatocellular carcinoma, radiotherapy was used with reference to orbital floor metastases to achieve a rapid palliative effect.

## Conclusions

Radiotherapy for ocular metastases is an important option, providing rapid symptomatic palliation even when the primary lesion is difficult to treat. This report highlights the significance of recognizing rare symptoms like ocular metastasis and adopting a tailored, multidisciplinary treatment approach for advanced HCC patients.
